# Competency in trauma surgery: a national survey of trainees and consultants

**DOI:** 10.1007/s11845-022-03117-4

**Published:** 2022-08-15

**Authors:** Michael Flanagan, Jessica Ryan, Gary Bass, Kevin Barry, Oscar Traynor, Morgan McMonagle

**Affiliations:** 1 Department of Surgery, University Hospital Waterford, Waterford, Ireland; 2 Division of Traumatology, Surgical Critical Care and Emergency Surgery, Penn Medicine, Philadelphia, PA USA; 3 Department of Surgical Affairs, National Surgical Training Programmes, Royal College of Surgeons in Ireland, Dublin, Ireland; 4 Department of Surgical Affairs, Royal College of Surgeons in Ireland, Dublin, Ireland; 5 Imperial College London, London, UK

**Keywords:** Intercollegiate Surgical Curriculum Programme, Thoracotomy, Trauma skills, Trauma surgery

## Abstract

**Background:**

The current sparsity of surgical trainees’ exposure to training in operative trauma surgery is multifactorial. This concern has been addressed in the revised Intercollegiate Surgical Curriculum Programme (ISCP) for general and vascular surgery (2021). In the lead up to its implementation, we aimed to assess both trainee and consultant confidence levels as a surrogate reflection in the core competency operative skills in general emergency trauma surgery, identify individual experience in commonly performed trauma procedures and gauge interest in a career in trauma surgery.

**Method:**

An online survey was circulated to general surgery and vascular surgery trainees and consultants. Self-reported competencies were assessed using a 1–10 confidence rating scale. Most questions were based on competencies in emergency trauma surgery as set out by the ISCP.

**Results:**

Out of 251 surgical trainees and consultants, 119 responded to our survey (47.4% response rate). Less than half (44.1%; *n* = 52) of respondents had experienced a trauma thoracotomy. Respondents scored ‘somewhat’ or ‘not at all’ competent in the majority of competencies assessed.

**Conclusion:**

Self-reported competencies in operative trauma skills across all subgroups were sub-standard with incremental levels of perceived competence proportional to years of surgical training. Our data supports the necessity of the new curriculum, in addition to modern training pathways with direct exposure to operative trauma surgery involving dedicated trauma centres and networks, and responsibility of training pathways in the provision of training trauma surgery.

**Supplementary Information:**

The online version contains supplementary material available at 10.1007/s11845-022-03117-4.

## Introduction

The intercollegiate surgical curriculum programme for general surgery has been recently revised and updated with added emphasis on emergency general surgery and trauma care, reflecting the growing need to train and upskill surgeons in the generality of emergency surgery [1]. This need is partly in response to the success of the UK Trauma Network [2] and is particularly relevant to Ireland as the new trauma service awaits implementation [3]. There are justifiable concerns amongst both trainees and trainers with regard to the lack of exposure to emergency surgery, particularly trauma with a resultant potential for substandard competencies in operative trauma procedures that may be encountered as a newly appointed consultant surgeon. This is in part due to the increasingly non-operative nature of injury management [4], a reduction in surgical exposure associated with the European Working Time Directive [5] and a failure to establish high-volume centralised major trauma care in certain jurisdictions that would to facilitate focused training pathways in operative trauma skills [6].

To better understand current competencies in trauma surgery among trainees and consultants, both general and vascular surgery trainees and consultants were surveyed on a broad range of competencies in trauma surgery as prescribed by the new curriculum. The aim of this study was to assess the individuals’ confidence level as a surrogate reflection of competency level in general emergency trauma surgery prior to commencement of the new Intercollegiate Surgical Curriculum Programme [1]. In addition, we wanted to gauge interest in the speciality of trauma surgery among trainees and consultants, which may inform stakeholders in potential workforce arrangements.

## Methods

### Participants

Data was collected between 31 January and 5 February 2021 using an anonymised online survey (SurveyMonkey®, SurveyMonkey Inc., San Mateo, CA). Specialty training administrators working at the Royal College of Surgeons in Ireland (RCSI) assisted with the survey distribution to relevant consultant surgeons and trainees to ensure General Data Protection Regulation (GDPR) compliance and using their respective Internet Protocol addresses only. Two-hundred fifty-one surgeons were contacted (100 general surgery consultants, 76 general surgery trainees, 34 vascular consultants and 50 vascular trainees), via email with an invitation to participate including an explanation of the survey with the relevant link. Completion of the survey was interpreted as informed consent, and no compensation was offered in reward for participation. This study has been reported in line with the Strengthening the reporting of cohort, cross-sectional and case–control studies in surgery (STROCSS) criteria [7].

### Survey questions

Appendix 1 outlines the survey questions posed. Demographics data collected included age, gender, subspecialty and country of current practice. Information on level of training was also collected; including years since graduation, training stage and years since appointment at consultant grade. For training stage, the UK and Irish grading system was used, which comprises specialist trainees (levels ST3 through to ST8), non-consultant post-specialist training and consultant level.

Self-reported competencies were assessed using a 1–10 confidence rating scale (Table 1). A larger 10-point Likert scale was used to increase the variance as compared to smaller 7-point or 5-point Likert scales, to obtain a higher degree of measurement precision and better detect changes in sequential responses among the respondents [8]. Questions were developed using a selection of 26 technical skill-based competencies taken from the most recent General Surgical Curriculum from the ISCP [1]. Additionally, participants were asked if they had ever been involved in a trauma thoracotomy, and its location (emergency department/trauma bay or operating theatre). The full list of questions can be found in Appendix 1. Finally, participants were asked whether they felt that Trauma and Emergency Surgery should be considered a separate subspecialty within surgical training, and whether or not they themselves were interested in being a Trauma and Emergency surgeon.

**Table 1:** The 1–10 competency rating scale

Score	Competency
1	Not at all competent
2–4	Somewhat competent
5–7	Quite competent
8–10	Highly competent

### Data analysis

Results were analysed using Predictive Analytics Software (PASW 18.0.2, SPSS Inc., Chicago, IL, USA). Descriptive data are presented as absolute frequencies and percentages. Continuous data are presented as means and standard deviations. For analysis, pre-CCST (ST3-8) and post-CCST (post-CCST non-consultant, consultant) respondents were grouped together and compared. Comparative analyses of quantitative data were performed using Student’s *t*-test for continuous variables. All tests of significance were 2-tailed, with *p* < 0.05 indicating statistical significance.

## Results

### Demographics

A total of 119 surgical trainees and consultants responded, giving a response rate of 47.4%. One third were female (*n* = 76), and the majority (*n* = 71/114; 61.7%) had graduated more than 10 years prior (Table 2). A total of 63 out of 119 respondents (52.9%) were specialist trainees (ST3–ST8), and the remainder had completed their training. Of the consultant-level respondents, the majority (*n* = 41/51; 80.4%) had been in practice for greater than 5 years. Colorectal and vascular surgery respondents accounted for 43/115 (37.4%) and 30/115 (26.1%) of subspecialties, respectively (Table 3). Almost all (95.6%) respondents were practicing in Ireland at the time of the survey.

**Table 2:** Participant demographics

	*N* (%)
Gender	
Male	76 (66)
Female	36 (31.3)
Prefer not to say	3 (2.6)
Years since graduation	
< 5	8 (6.9)
5–9	35 (31.3)
10–14	21 (18.3)
15–20	15 (13)
> 20	35 (30.4)
Stage of training	
ST3–5 or equivalent	40 (33.6)
ST6–8 or equivalent	23 (19.3)
Post-CCST non-consultant	5 (4.2)
Consultant	51 (42.9)
< 5 years appointed	10 (19.6)
> 5 years appointed	41 (80.4)

*CCST* Certificate of Completion of Specialist Training, *ST* year of surgical training

**Table 3:** Subspecialty by level of training

		*N* (%)		
Subspecialty	Pre-CCST		Post-CCST	Total
Upper gastrointestinal/bariatric	7 (11.9)		3 (5.4)	10 (8.7)
Hepatobiliary	3 (5.1)		1 (1.8)	4 (3.5)
Colorectal	23 (39.0)		20 (35.7)	43 (37.4)
Breast/endocrine	8 (13.6)		10 (17.9)	18 (15.6)
Vascular	13 (22)		17 (30.4)	30 (26.1)
General/trauma/emergency	5 (8.5)		5 (8.9)	10 (8.7)

*CCST* Certificate of Completion of Specialist Training

### Trauma experience

Less than half (*n* = 52/118; 44.1%) of respondents had experienced a trauma thoracotomy, and of those that did only 25 of 52 respondents (48.1%) had experienced an emergency department/trauma bay thoracotomy. Only 15 of 62 (24.2%) trainees had any experience of a trauma thoracotomy. A total of 75 of 116 respondents (64.6%) felt that trauma and emergency surgery should be considered a separate subspecialty within general surgery training. A total of 36 of 116 respondents (31.3%) expressed an interest in Trauma and Emergency surgery as their subspecialty interest of choice.

Of the 26 competencies surveyed, the majority of respondents scored as ‘somewhat’ or ‘not at all’ competent, with only 10 reporting a mean score of 5 or greater. No competency had a mean score consistent with a high degree of competence (i.e. 8–10) with that particular injury or skill (Table 1). The lowest scoring (score < 3) competencies were head of pancreas resection for trauma, management of a retrohepatic IVC injury, repair of an injured kidney and management of a combined pancreatico-duodenal injury. The highest scoring (score > 7) competencies were management of a bowel injury (both large and small bowel) and trauma laparotomy (Table 4). When pre- and post-CCST respondents were compared, post-CCST respondents scored significantly higher in every self-reported competency (Table 5). Scores of the post-CCST respondents were sub-standard in a number of self-reported trauma competencies.

**Table 4:** Self-reported competency in the surgical management of an unstable trauma patient

		*N* (%)				
		Not at all competent	Somewhat competent	Quite competent	Highly competent	Mean competency score (SD)
1	Performing a thoracotomy/clamshell thoracotomy	28 (28.3)	34 (34.3)^*^	27 (27.2)	10 (1)	3.7 (2.5)
2	Suturing the heart/cardiac repair for trauma	35 (35.3)^*^	35 (35.3)^*^	23 (23.2)	6 (6.1)	3.1 (2.3)
3	Performing a trauma laparotomy	2 (2.1)	14 (14.4)	30 (30.9)	51 (52.5)^*^	7.1 (2.4)^**^
4	Performing a blind trauma laparotomy	6 (6.2)	16 (16.5)	36 (37.1)	39 (40.2)^*^	6.4 (2.5)^**^
5	Packing the abdomen for major traumatic haemorrhage	4 (4.1)	16 (16.3)	32 (32.6)	46 (46.9)^*^	6.7 (2.4)^**^
6	Managing a small-bowel injury for trauma	4 (4.1)	9 (9.2)	23 (23.7)	61 (62.9)^*^	7.6 (2.5)^**^
7	Managing a large-bowel injury for trauma	5 (5.1)	10 (10.2)	25 (25.5)	58 (59.2)^*^	7.4 (2.7)^**^
8	Managing a rectal injury for trauma	9 (9.1)	25 (25.2)	27 (27.2)	38 (38.3)^*^	6 (3.1)^**^
9	Managing a gastric injury for trauma	8 (8.2)	21 (21.4)	39 (39.8)^*^	30 (30.6)	5.8 (2.5)^**^
10	Performing a trauma splenectomy	6 (6.1)	17 (17.3)	27 (27.5)	48 (49)^*^	6.6 (2.7)^**^
11	Packing an injured liver	5 (5)	22 (22.2)	41 (41.4)^*^	31 (31.3)	6 (2.5)^**^
12	Extraperitoneal pelvic packing via laparotomy	14 (14.4)	30 (30.9)	34 (35)^*^	19 (19.6)	5 (2.7)^**^
13	Extraperitoneal pelvic packing via pelvis only	20 (20.4)	34 (34.7)^*^	31 (31.6)	13 (13.3)	4.3 (2.7)
14	Performing a trauma nephrectomy	28 (28.6)	40 (40.8)^*^	20 (20.4)	10 (10.2)	3.6 (2.6)
15	Performing repair of an injured kidney (e.g. mesh repair)	47 (47.9)^*^	36 (36.7)	11 (11.2)	4 (4.1)	2.5 (2)
16	Operative management of a pancreatic injury	36 (36.4)	40 (40.4)^*^	16 (16.2)	7 (7.1)	3 (2.3)
17	Tail of pancreas resection for trauma	30 (30.6)	33 (33.7)^*^	25 (25.5)	10 (10.2)	3.7 (2.7)
18	Head of pancreas resection for trauma	62 (63.9)^*^	23 (23.7)	9 (9.2)	3 (3.1)	2.1 (2)
19	Managing a duodenal injury for trauma	19 (19.2)	41 (41.4)^*^	28 (28.3)	11 (11.1)	4.1 (2.5)
20	Managing a combined pancreatico-duodenal injury for trauma	43 (43.4)^*^	42 (42.4)	10 (10.1)	4 (4)	2.6 (2.1)
21	Retroperitoneal exposure for trauma	14 (14.1)	46 (46.5)^*^	27 (27.2)	12 (12.1)	4.2 (2.6)
22	Operative management of an infrarenal IVC injury	28 (28.6)	37 (37.7)^*^	22 (22.4)	11 (11.2)	3.6 (2.7)
23	Operative management of a retrohepatic IVC injury	49 (50)^*^	36 (36.7)	11 (11.2)	2 (2)	2.4 (1.8)
24	Operative management of a suprarenal aortic injury	39 (40.6)^*^	32 (33.3)	18 (18.7)	7 (7.3)	3 (2.3)
25	Operative management of a infrarenal aortic injury	23 (23.7)	34 (35)^*^	23 (23.7)	17 (17.5)	4.2 (2.8)
26	Performing a vascular shunt for arterial trauma	35 (35.7)^*^	31 (31.6)	13 (13.3)	19 (19.4)	3.6 (3)

^*^Highest percentage allocation; ^**^Mean score rated as quite competent or above

**Table 5:** Self-reported competency in the surgical management of an unstable trauma patient: a comparison between pre-CCST and post-CCST

		Pre-CCST	Post-CCST	*p* Value
		Mean (SD)		
1	Performing a thoracotomy/clamshell thoracotomy	2.73 (1.89)	4.57 (2.72)	< 0.001
2	Suturing the heart/cardiac repair for trauma	2.21 (1.63)	4.02 (2.53)	< 0.001
3	Performing a trauma laparotomy	6.09 (2.01)	8.04 (2.34)	< 0.001
4	Performing a blind trauma laparotomy (i.e. in the absence of imaging)	5.13 (2.21)	7.61 (2.23)	< 0.001
5	Packing the abdomen for major traumatic haemorrhage	5.32 (2.25)	8 (1.86)	< 0.001
6	Managing a small-bowel injury for trauma	6.52 (2.53)	8.67 (1.99)	< 0.001
7	Managing a large-bowel injury for trauma	6.19 (2.62)	8.52 (2.15)	< 0.001
8	Managing a rectal injury for trauma	4.73 (2.71)	7.18 (2.94)	< 0.001
9	Managing a gastric injury for trauma	5.06 (2.33)	6.6 (2.52)	< 0.001
10	Performing a trauma splenectomy	5.1 (2.37)	8 (2.18)	0.003
11	Packing an injured liver	4.9 (2.22)	6.98 (2.38)	< 0.001
12	Extraperitoneal pelvic packing via laparotomy	3.4 (1.98)	6.55 (2.37)	< 0.001
13	Extraperitoneal pelvic packing via pelvis only	3 (1.91)	5.58 (2.67)	< 0.001
14	Performing a trauma nephrectomy	2.63 (1.86)	4.52 (2.83)	< 0.001
15	Performing repair of an injured kidney (e.g. mesh repair)	1.79 (1.1)	3.1 (2.41)	< 0.001
16	Operative management of a pancreatic injury	2.13 (1.56)	3.84 (2.6)	< 0.001
17	Tail of pancreas resection for trauma	2.67 (1.83)	4.66 (2.99)	< 0.001
18	Head of pancreas resection for trauma	1.54 (1.21)	2.63 (2.43)	0.007
19	Managing a duodenal injury for trauma	3.15 (2)	4.98 (2.69)	< 0.001
20	Managing a combined pancreatico-duodenal injury for trauma	1.88 (1.39)	3.22 (2.36)	0.001
21	Retroperitoneal exposure for trauma	3.02 (1.84)	5.29 (2.68)	< 0.001
22	Operative management of an infrarenal IVC injury	2.23 (1.62)	5 (2.76)	< 0.001
23	Operative management of a retrohepatic IVC injury	1.68 (1.15)	2.98 (2.12)	< 0.001
24	Operative management of a suprarenal aortic injury	1.91 (1.37)	3.98 (2.61)	< 0.001
25	Operative management of a infrarenal aortic injury	3.06 (2.24)	5.3 (2.92)	< 0.001
26	Performing a vascular shunt for arterial trauma	2.79 (2.49)	4.47 (3.32)	0.006

*CCST* Certificate of Completion of Specialist Training. The level of significance is *p* < 0.05, and all *p* values reached significance

## Discussion

This study demonstrates incremental levels of perceived competence in operative trauma skills proportional to the years of surgical training and into consultant-level practice. Higher levels of competency were reported for those procedures that are mirrored with similar routine elective or non-trauma emergency work, such as small-bowel resection, with lower levels of competency for the more severe injuries or those not routinely performed outside of the specialty of trauma surgery (e.g. thoracotomy).

Less than half of respondents reported having performed or been involved in a trauma thoracotomy, which is an expected competency skill for exsanguinating injury, especially penetrating chest trauma. A total of 15 out of 62 respondents involved in a trauma thoracotomy (24.2%) were of training grade only. A trauma thoracotomy is a life-saving skill in which all surgeons taking acute call should be competent, in addition to the complex decision making around it, as reflected in the new ISCP curriculum. Recent analysis of registry data on resuscitative thoracotomy identified that in 76.8% (109/142) of cases the procedure was performed in the emergency department [9], further highlighting the need for surgical expertise and appropriate training. Guidelines and consensus statement documents outline the accepted and selective indications for which emergency department thoracotomy is appropriate and indications for which survival is poor, e.g., blunt trauma [10, 11].

As predicted, trainees are lacking in competence for those procedures that they are less frequently exposed to, yet many of these could be described as ‘expected skills’ for a newly appointed surgeon managing complex polytrauma as part of acute call. It is clear from our data that a significant deficiency in trauma operative skills exists for many of the injuries that a newly appointed consultant surgeon will encounter within a wider trauma network or major trauma centre. This is especially concerning for those practicing in Ireland, where centralisation of trauma services has yet to be implemented. Therefore surgeons without sub-specialty knowledge and skill in trauma surgery are much more likely to be deficient in the management of complex polytrauma.

Competence in certain key trauma procedures requires development during training and must be maintained throughout consultant practice for all surgeons taking acute care admissions. The revised ISCP curriculum acknowledges this and that certain skills and capabilities are best learned and maintained within the formal setting of a specific taught course (e.g. general management of the multiply injured patient). There is only one mandated course in the revised curriculum [1] but multiple other highly recommended courses are available. Acquiring certain clinical trauma skills to the level of competency sufficient to manage severe injury is challenging due to its infrequency. Successful course completion that has a high level of governance associated with it may be invaluable. Examples of courses that meet the required learning outcomes as outlined by ISCP include the Definitive Surgical Trauma Skills (DSTS) course, Advanced Trauma Life Support (ATLS), and the European Trauma Course. However, only one of these (DSTS) teaches advanced operative trauma skills and the associated complex decision making required.

The role of simulation in addressing the paucity of trauma operative training has also been addressed within the revised ISCP curriculum. A recent survey of UK trainees has proposed the introduction of a live large animal course to facilitate trauma training [12]. Among 54 trainee respondents to the survey, 90% agreed or strongly agreed that a live large animal course with exposure to major intraoperative haemorrhage is beneficial. However, the ethics and costs that come with such a course may be prohibitive. Cadevaric dissection provides a high-fidelity training experience incorporating equipment, environment and behavioural factors that are difficult to replicate in a synthetic model although evidence currently does not demonstrate superiority over lower fidelity models [13].

A number of developments are underway to address competencies in key trauma operative skills. The implementation and rollout of the new ISCP Curriculum aims to re-establish “Emergency/Elective competencies across the generality of surgery” as a primary focus during higher specialist training. The successful reconfiguration of trauma care within the UK [2] and that planned in Ireland [3] has implications on how trauma surgery training is provided and how a career in trauma surgery will mature going forward [3]. Having a defined and dedicated trauma network provides improved exposure to and training in the optimal management of the severely injured patient, including key operative damage control skills.

It is important to note the level of interest amongst the respondents in a career dedicated to the subspecialty of trauma and emergency surgery. Unlike the USA, there is currently no dedicated pathway for a unique career in trauma surgery in Ireland, notwithstanding attempts to address this in the UK, with the Training Interface Group (TIG) trauma fellowship. The TIG fellowships were established in 2019 to meet the increased demand for trauma-competent surgeons within the UK trauma network and as a pathway to acquire operative skills [4]. This training pathway includes both ‘resuscitative’ and ‘operative’ pathways to match the skills acquisition across a broad spectrum of training for dedicated personnel within the major trauma network.

There are a number of limitations to our current study. Vascular and general trainees and consultants were surveyed on a broad spectrum of trauma procedures, some of which are particular to subspecialty training and experience, potentially skewing results in favour of the particular consultant/trainee’s chosen field. Our chosen Likert type scale was not validated in a pilot study with a smaller cohort prior to circulation to the total consultant and trainee body [14, 15]. The author’s definition of competence was not shared with the survey respondent prior to survey completion and thus the respondents’ view of competence may differ from that of the author or other respondents. The use of the word competence and its relationship with confidence in performing a trauma procedure is difficult to either define or measure. A systematic review and qualitative discourse analysis to critically analyse language used to measure general surgery trainees’ confidence or readiness to practice concluded that robust methods of measuring self-efficacy be a primary focus rather than confidence or competence which are difficult to define and measure [16].

Our study is a snapshot survey of trainees and qualified surgeons regarding their competency/confidence managing a major trauma operative case load. The results of this nationwide survey of trauma competencies in consultants and trainees have wider implications for the provision of trauma surgery services. There are clearly concerns with regards to a deficiency of experience in our system to date, which is important to address ahead of the roll out of the major trauma network in Ireland. A number of means are suggested to address low levels of competency in trauma procedures, such as trauma simulation and trauma immersion in high-volume centres. It is likely that a solution will come from a combination of both. Over 30% of respondents reported that they would be interested in being a trauma surgeon. It is the responsibility of training bodies, key government stakeholders, and trainees to define the role and career pathway of the trauma surgeon in the provision of the trauma care.

## Supplementary Information

Below is the link to the electronic supplementary material.

Supplementary file1 (DOCX 64 KB)
