# Supplementary material for: Competency in trauma surgery: a national survey of trainees and consultants
Source: Ir J Med Sci. 2022 Aug 15;192(3):1303–9. doi: 10.1007/s11845-022-03117-4 (PMC10250435; doi:10.1007/s11845-022-03117-4)
Supplement: Supplementary file 1 — Supplementary file1 (DOCX 64 KB) [file 11845_2022_3117_MOESM1_ESM.docx]

#### 1. Age

<30

31-35

36-40

41-50

>50

2. Gender

Male

Female

prefer not to say

3. Years since graduation

<5

5-9

10-14

15-20

>20

4. Subspecialty

Upper GI/Bariatric

Hepatobiliary

Colorectal

Breast/Endocrine

Vascular

General/Trauma/Emergency

Other (please specify)

5. Stage of training

ST3-5 or equivalent

ST6-8 or equivalent

post CCST non-consultant

Consultant

 6. Have you ever been involved in a trauma thoracotomy

Yes

No

7. Have you ever been involved in a trauma thoracotomy in the Emergency Department/Trauma bay

Yes

No

## **Competency in the surgical management of an unstable trauma patient**

1-10 competency rating scale

**1**        not at all competent
**2-4**    somewhat competent
**5-7**    quite competent
**8-10**  highly competent

8. How competent do you feel performing a trauma thoracotomy/clamshell thoracotomy

#### 9. How competent do you feel suturing the heart/cardiac repair for trauma

#### 10. How competent do you feel performing a trauma laparotomy

#### 11. How competent do you feel performing a blind trauma laparotomy (i.e. in the absence of imaging)

#### 12. How competent do you feel packing the abdomen for major traumatic haemorrhage

#### 13. How competent do you feel managing a small bowel injury for trauma

#### 14. How competent do you feel managing a large bowel injury for trauma

#### 15. How competent do you feel managing a rectal injury for trauma

#### 16. How competent do you feel managing a gastric injury for trauma

#### 17. How competent do you feel performing a trauma splenectomy

#### 18. How competent do you feel packing an injured liver

#### 19. How competent do you feel at extraperitoneal pelvic packing via laparotomy

#### 20. How competent do you feel at extraperitoneal pelvic packing via pelvis only

#### 21. How competent do you feel performing a trauma nephrectomy

#### 22. How competent do you feel at performing repair of an injured kidney (e.g. mesh repair)

#### 23. How competent do you feel in the operative management of a pancreatic injury

#### 24. How competent do you feel at tail of pancreas resection for trauma

#### 25. How competent do you feel at head of pancreas resection for trauma

#### 26. How competent do you feel managing a duodenal injury for trauma

#### 27. How competent do you feel managing a combined pancreatico-duodenal injury for trauma

#### 28. How competent do you feel at retroperitoneal exposure for trauma

#### 29. How competent do you feel in the operative management of an infra renal IVC injury

#### 30. How competent do you feel in the operative management of a retro hepatic IVC injury

#### 31. How competent do you feel in the operative management of a supra renal aortic injury

#### 32. How competent do you feel in the operative management of a infra renal aortic injury

#### 33. How competent do you feel performing a vascular shunt for arterial trauma

34. Do you think Trauma and Emergency Surgery should be considered a separate subspecialty in surgical training

Yes

No

35. Would you be interested in being a Trauma and Emergency surgeon

Yes

No

36. Where is your current surgical practice

Ireland

U.K.

North America

Australia / NZ

Middle East

Other (please specify)
